# Supplementary material for: Antibody Responses to SARS-CoV-2 Infection—Comparative Determination of Seroprevalence in Two High-Throughput Assays versus a Sensitive Spike Protein ELISA
Source: Vaccines (Basel). 2021 Nov 11;9(11):1310. doi: 10.3390/vaccines9111310 (PMC8624239; doi:10.3390/vaccines9111310)
Supplement: Supplementary file 1 [file vaccines-09-01310-s001.zip › vaccines-1442118-supplementary.pdf]

1. Supplementary Data

Table S1. Summary of performance characterizes of each immunoassay as provided by the manufacturer.

**Assay and analyser used**	**Viral target and antibody type**	**Sample type**	**Sensitivity (95% CI) on samples taken ≥14 days post-symptom onset/post-RT-PCR positive, [sample numbers]**	**Specificity (95% CI), number of samples**	**Manufactures threshold value**
Abbott SARS-CoV-2 Immunoassay, Architect i2000SR	Nucleocapsid protein, IgG	Serum, serum separator tube and plasma.	100% (95.89-100), [88]	99.63% (99.05, 99.90), [1070]	Negative: <1.4 Positive: ≥1.4
Roche Elecsys® Anti-SARS-CoV-2, Cobas e 411	Nucleocapsid protein, IgG	Serum collected using standard sampling tubes. Li-heparin, K2-EDTA and K3-EDTA Plasma	99.5% (97.0-100%), [496]	99.80% (99.69, 99.89%), [10,453]	Non-reactive: <1.0 Reactive: ≥1.0
The Binding Site Anti IgG/A/M SARS-CoV-2 ELISA, Dynex DS2	S1/S2 spike protein, Total antibody	Serum, serum separating tube.	98.6 % (92.6-100), [162]	98.3 (96.4-99.4), [707].	Negative: <1.0 Positive: ≥1.0,
